# Enhanced meaning in life following psychedelic use: converging evidence from controlled and naturalistic studies

**DOI:** 10.3389/fpsyg.2025.1580663

**Published:** 2025-06-06

**Authors:** William Roseby, Hannes Kettner, Leor Roseman, Meg J. Spriggs, Taylor Lyons, Joe Peill, Bruna Giribaldi, David Erritzoe, David J. Nutt, Robin L. Carhart-Harris

**Affiliations:** ^1^Centre for Psychedelic Research, Department of Brain Sciences, Faculty of Medicine, Imperial College London, London, United Kingdom; ^2^Carhart-Harris Lab, Department of Neurology, University of California, San Francisco, San Francisco, CA, United States

**Keywords:** psychedelics, psilocybin, mystical experience, meaning, meaning in life, depression, wellbeing

## Abstract

**Introduction:**

Psychedelics, such as psilocybin, are increasingly recognized for their propensity to elicit powerful subjective experiences that carry personal meaning. While research has demonstrated the capacity for these compounds to promote psychological wellbeing, it has yet to be shown to what extent they modulate "meaning in life", a specific contributor to mental and physical health.

**Methods:**

Using the Meaning in Life Questionnaire (MLQ), we examined changes in meaning in life occurring across three different contexts of psychedelic use, including a randomized clinical trial of psilocybin for depression, controlled administration of psilocybin in a single-arm healthy volunteer study, and a naturalistic observational study following participants in psychedelic retreats. Meaning in life changes were analyzed with linear mixed models, and relationships to other predictors and outcomes were examined via Pearson correlations.

**Results:**

Across all contexts, the sub-factor “presence of meaning” was strongly increased after a psychedelic experience, while the sub-factor “search for meaning” was only weakly reduced. Enhancements of meaning in life were also moderately correlated with changes in measures of mental health, including mental wellbeing and depression severity. In line with previous research, we found that mystical, ego dissolution and emotional breakthrough experiences were correlated with an increase of meaning in life, with context-dependent differences in the strength of the association.

**Discussion:**

The convergence of evidence from multiple studies shows that psychedelic use has a robust and long-lasting positive effect on meaning in life. We explore potential mechanisms of psychedelic-induced meaning enhancement and highlight the possible influences of psychosocial context on outcomes.

## 1 Introduction

Since their initial emergence into Western culture in the 1950s, classical psychedelics like psilocybin and LSD have been recognized for their ability to produce profound subjective experiences (Pahnke and Richards, 1966; Pahnke, 1969; Griffiths et al., 2018). Recent research has shown that psychedelic experiences have a reliable capacity for inducing long-term improvements in mental wellbeing (Haijen et al., 2018; Mans et al., 2021) and for the treatment of mood (Carhart-Harris et al., 2016a; Griffiths et al., 2016; Carhart-Harris et al., 2021) and addictive disorders (Zafar et al., 2023). Several lines of research have demonstrated that the intensity of acute subjective effects experienced following psychedelic intake are associated with beneficial psychological outcomes, with the greatest amount of evidence related to so-called mystical-type experiences characterized by a perception of blissful oneness (Haijen et al., 2018; Roseman et al., 2018; Weiss et al., 2024). The subjective profundity of the psychedelic mystical experience is such that it is often rated among the most meaningful and spiritually significant of an individual's life (Griffiths et al., 2006, 2008; Gukasyan et al., 2022). These reports have led to suggestions that psychedelics act to enhance the perception of meaning (Hartogsohn, 2018), and that this may be a core component of their therapeutic mechanism, particularly when combined with psychological support (Nayak and Johnson, 2021). However, the recently renewed period of psychedelic research has yet to properly assess the impact of psychedelics on meaning in life.

The definition of “meaning in life” has been subject to debate (Park, 2010; Martela and Steger, 2016; King and Hicks, 2021), but it has most frequently been linked to the concept of eudaimonia: a sense of fulfillment arising the actualisation of long-term goals (Steger and Frazier, 2005). Perhaps the best-known discussion on meaning is found in Viktor Frankl's seminal 1946 work *Man's Search For Meaning*, in which he detailed his own experience as a Holocaust survivor. Frankl posited that creating meaning was the primary drive behind human behavior, and that being able to hold meaning—even in extremely brutal and dehumanizing circumstances—served as a protective factor for mental and physical health. More contemporary evaluations of meaning in life have produced a three-dimensional model comprising (1) coherence, the degree to which individuals feel they can make sense of the world, (2) purpose, the commitment to long-term goals, and (3) significance, the extent to which people regard their lives as valuable (Martela and Steger, 2016; King and Hicks, 2021). Various conditions and events also serve to create and alter meaning over time. Ample research suggests that meaning is made when pre-existing beliefs are challenged, particularly through adverse life events (Park, 2010), while some research has specifically suggested that individuals create meaning to counteract anxiety surrounding death (Simon et al., 1998; Steger and Frazier, 2005). Regardless of its role, meaning in life—and closely related constructs like Purpose and the Japanese concept of Ikigai—have been repeatedly linked with both mental and physical health (King and Hicks, 2021; Wilkes et al., 2022). Higher ratings of meaning in life have been associated with increased subjective wellbeing (Zika and Chamberlain, 1987) and reduced levels of anxiety and depression (Debats, 1996), while a 2017 meta-analysis found small but significant positive correlations between meaning in life and objective measures of health (Czekierda et al., 2017). Conversely, over-commitment to searching for meaning in life has overall been associated with negative affect, including increased ratings of anxiety and depression (Steger et al., 2006). Altogether, meaning in life presents a target for long-term overall health that is yet distinct from other measures of psychological wellbeing (Steger et al., 2006); a target that could be particularly enhanced by psychedelics as compared to other pharmacological interventions (Hartogsohn, 2018).

The contemporary hypothesis that psychedelics enhance personal meaning follows an integration of recent psychological and neuroscientific inquiries. From a psychological perspective, the psychedelic state can present a challenging element, often associated with the perceived loss of one's sense of self, termed ego-dissolution (Barrett et al., 2016; Nour et al., 2016). However, many who undergo this type of experience still report it as beneficial, if they are able to integrate it into a life narrative (Carbonaro et al., 2016). This process parallels the research on meaning-making in response to adverse life situations (Park, 2010). Furthermore, the sheer intensity of the mystical-type experience compared to everyday consciousness could serve as a prominent memory around which meaning is created, as suggested by studies showing associations between mystical experience and lasting personality change (Nour et al., 2017; Kettner et al., 2019). The process of meaning-making may also be expedited by the emotional cartharsis of the psychedelic state, which can allow movement through difficult and previously unprocessed emotional states (Roseman et al., 2019), long-considered a key aspect of psychological healing (Greenberg, 2012). The rapid belief change provoked by psychedelics is explicated by a variety of neuroimaging studies, which point to a model of acutely enhanced neuronal flexibility. For example, fMRI studies on acute psychedelic effects have indicated increased global integration of the brain (Tagliazucchi et al., 2016), alongside specific disintegration of the default mode network (DMN), a brain network related to rumination and sense of self (Carhart-Harris et al., 2017; Gattuso et al., 2023). Both of these effects have been observed to correlate with the subjective experience of mystical experience and ego-dissolution (Carhart-Harris et al., 2017; Tagliazucchi et al., 2016). Meanwhile, EEG studies have reported increases in brain entropy in the psychedelic state (Timmermann et al., 2019; Mediano et al., 2024). These findings have led to unifying theories of psychedelic action where increased brain entropy relaxes high-level beliefs, providing an opportunity for reframing entrenched perspectives (Carhart-Harris et al., 2014; Carhart-Harris and Friston, 2019; Zeifman et al., 2025). While neuroimaging studies on meaning in life are limited, there is some evidence that changes in DMN functional connectivity are also involved (Waytz et al., 2015; Mwilambwe-Tshilobo et al., 2019). Thus, the acute state of psychedelic-induced neuronal and cognitive flexibility (Daws et al., 2022; Lyons et al., 2024) could be particularly beneficial for re-evaluation of meaning in life.

The aim of the present study was to investigate changes in meaning in life after a psychedelic experience. While some previous studies have indicated potential increases in ratings of meaning in life following psychedelic use (Mans et al., 2021; Timmermann et al., 2024; Erritzoe et al., 2024), as well as related changes including greater meaning in the world (Nayak et al., 2023), and purpose in life (Griffiths et al., 2016), these have lacked investigation of contextual factors that are especially influential to psychedelic outcomes (Carhart-Harris et al., 2018; Nayak et al., 2023). Therefore, we compiled and compared data from several studies that included naturalistic and controlled settings, as well as populations of healthy psychedelic-naive individuals, patients with clinical depression, and a large group of ceremony attendees. Meaning in life was measured using the Meaning in Life Questionnaire (MLQ), which comprises “presence” (MLQ-P) and “search” (MLQ-S) subscales (Steger et al., 2006). In line with the other positive effects of psychedelics, we formulated three hypotheses. H1: the presence of meaning in life will be increased following a psychedelic experience, while search for meaning will be attenuated; H2: changes in meaning in life will be correlated with changes in depression severity and overall wellbeing; and H3: positive changes in meaning in life will be correlated with acute qualities of the psychedelic experience including mystical-type experiences.

## 2 Methods

### 2.1 Psychometrics

To assess changes in outcome measures including meaning in life, we employed validated rating scales designed to measure underlying psychological traits. We also took this approach to assess qualities of the acute psychedelic state, using scales that probe experience states. Although the individual studies employed a range of measures, we mention below only those pertinent to the present analysis.

#### 2.1.1 Meaning in life questionnaire

The MLQ was developed by Steger and colleagues in 2006 to assess perceived meaning in life (Steger et al., 2006). It comprises 10 items rated on a 7-point Likert scale, where 1 is “Absolutely Untrue” and 7 is “Absolutely True.” The 10-item form was validated across three studies, and contains a two factor structure to measure the presence of (MLQ-P) and search for (MLQ-S) meaning in life. The two factors are typically somewhat inversely correlated (*r* = −0.19), and each overlap with other wellbeing-related measures such as Life Satisfaction, Neuroticism, and Depression, where Presence is associated with positive wellbeing and Search with negative wellbeing. However, in comparison to previous measures of meaning in life such as the Purpose in Life Index and the Life Regard Index, the MLQ shows a reduced level of shared variance with these other well-being measures, as well as evidence of increased discriminant validity (Steger et al., 2006). Thus, the MLQ presents the current best rating scale to distinctly assess meaning in life as separate from similar, but related wellbeing constructs. The full questionnaire can be found at Supplementary Material Table S1.

#### 2.1.2 Warwick-Edinburgh mental wellbeing scale

The WEMWBS was developed and validated by Tennant and colleagues in 2007 to assess general mental wellbeing (Tennant et al., 2007). It contains 14 items rated on a 5-point Likert scale where 1 is “none of the time” and 5 is “all of the time.” The items are directed at several aspects of mental wellbeing such as stress, clarity and social connection, but ultimately converge on a single unified factor of mental well-being. It has been used previously to assess changes in psychological health following psychedelic use (Haijen et al., 2018; Peill et al., 2022), including in major depression (Carhart-Harris et al., 2021) and in the case of negative responders to psychedelics (Marrocu et al., 2024). Here, this scale was employed to investigate the association between general mental wellbeing and meaning in life following psychedelic intake.

#### 2.1.3 Beck depression inventory

The BDI was first published in 1961 by Beck and colleagues with the aim of standardizing the measurement of depressive symptomatology (Beck et al., 1961). Subsequent revisions (IA and II) have been developed to increase reliability and better align with diagnostic criteria (Beck et al., 1996). The scale, in the IA form, was employed as a secondary outcome measure in a trial of escitalopram against psilocybin for depression, although this measure showed greater differences between the treatment groups compared to the primary outcome measure (Carhart-Harris et al., 2021). The BDI-IA contains 21 items, each rated from 0 to 3 based on the severity of symptoms over the past two weeks; total scores range from 0 to 63, with higher scores indicating more severe depression (Beck et al., 1996).

#### 2.1.4 Mystical experience questionnaire

Originally developed by Pahnke in the 1960s to measure the occurrence of acute mystical experiences occasioned by psilocybin, the MEQ has been more recently revised by Maclean and colleagues to include 30 items that separate into five distinct factors: Mystical, which assess feelings of unity and insight; Positive mood, which measures feelings of joy and euphoria; Transcendence of time/space, which assess feelings of dimensional distortions; and Ineffability, which captures the extent to which the experience cannot be described (Maclean et al., 2012). The scale has been subsequently validated in experimental studies with psilocybin (Barrett et al., 2015). Total summed scores range from 0 to 150, while scores for the sub-factors total 15 (Ineffability), 30 (Positive mood, Transcendence of time/space), and 75 (Mystical).

#### 2.1.5 Ego-dissolution inventory

The EDI was developed and validated by Nour et al. (2016) to measure the perceived loss of ego during psychedelic experiences. It comprises 8 items rated on a visual analog scale from 0–100, where 0 is “No, not more than usually,” and 100 defined as “Yes, entirely or completely.” The items were found to target one single factor, which correlates strongly with the unitive mystical experience as measured via the MEQ. However, the scale also presents discriminant validity for perceptions of ego-inflation, which can occur during some types of mystical experience (Nour et al., 2016). Summed scores range from 0 to 800.

#### 2.1.6 Emotional breakthrough inventory

The EBI was developed and validated by Roseman et al. in 2019 to measure the extent of emotional catharsis during psychedelic experiences (Roseman et al., 2019). It contains 6 items rated on a visual analog scale from 0–100, where 0 is “No, not more than usual” and 100 is “Yes, entirely or completely.” The 6 items converge on a single factor, which is related to but distinct from mystical experiences as assessed with the MEQ (Roseman et al., 2019). Subsequent use of the scale in survey and laboratory studies have demonstrated its value for predicting wellbeing outcomes (Peill et al., 2022), as well as a positive association with preparedness for a psychedelic experience (McAlpine et al., 2024). Total summed scores range from 0 to 600.

### 2.2 Data acquisition

To assess meaning in life across various contexts, data was acquired from three previous studies that differed in their sample characteristics, drug of use, and the setting of the psychedelic experience. Each of these samples are described in more detail below, and are summarized in Table 1.

**Table 1:** Study design for the three samples analyzed in this study.

**Sample**	**Study design**	**Drug and dose**	**Timing**	**Measures**	**Sample**
Ceremony	Prospective online survey of ceremonial psychedelic experiences.	Ayahuasca (DMT) or psilocybin fungi; variable dose.	Baseline: 2 weeks prior; Follow-ups: 2 and 4 weeks, 6 months post.	MLQ, WEMWBS, MEQ, EDI, EBI	886 respondants.
Insight	One-arm, fixed order, within subjects lab study.	Psilocybin; 1 mg placebo, 25 mg active dose.	Placebo dose followed by active dose at 4 weeks. Baseline: one day before dosing. Follow-up: 2 weeks post.	MLQ, WEMWBS MEQ, EDI EBI	28 healthy participants.
Psilodep	Two-arm, active comparator, double- blind, randomized controlled, betweensubjects lab trial.	Psilocybin; 1 mg placebo for escitalopram group, 25 mg for psilocybin group.	2 dosing sessions 3 weeks apart; Baseline: one day before first dose; Follow-up: 6 weeks.	MLQ, BDI MEQ, EBI EDI	59 patients with major depressive disorder; 30 in psilocybin group, 29 in escitalopram group.

#### 2.2.1 Ceremony study

The Ceremony Study was an online prospective study in which participants planning to attend a naturalistic ceremonial retreat completed questionnaires prior to and following the retreat, which involved one to three psychedelic ceremonies. Responses were collected two weeks prior to the retreat (baseline), and at one day, 2 weeks, 4 weeks, and 6 months post-ceremony. Outcome measures, the MLQ and WEMWBS, were collected at baseline, 4 weeks and 6 months. Acute experience measures including the MEQ, EDI, and EBI, were collected at one day post-experience(s); the maximum value of these measures was taken for analysis since the strongest subjective experience was predicted to have the greatest impact on outcome measures. The survey attracted 886 participants in total. Depending on the ceremony, participants either consumed psilocybin or the traditional South American brew ayahuasca, which contains the classic psychedelic dimethyltryptamine (DMT). Ceremonies were conducted in a group setting with a guide and a generally supportive environment. Primary results from this study with a smaller sample were published in Kettner et al. (2021), while the current sample was later published in Peill et al. (2022).

#### 2.2.2 Insight

The Insight study was a laboratory study of the long-term effects of psilocybin on psychology and neurophysiology in healthy, psychedelic-naive participants. The participants completed two psilocybin dosing sessions separated by one month, with follow-ups at 2 weeks following each session. In the first session, participants received an inactive 1 *mg* placebo dose, while in the second session they received an active 25 *mg* dose; the subjects were blind to what dose they received. 28 participants completed the study in total, although MLQ scores were not acquired for all participants at all time points. The setting during the dosing sessions was therapeutic and psychologically supported by experienced guides. In the present study, we compared outcomes for the 1 *mg* and 25 *mg* doses: for the 1 *mg* inactive condition, we compared baseline scores to scores at 2 weeks after the first dose, while for the 25 *mg* active condition we compared scores at 4 weeks — just before the second dose—to scores at 6 weeks—2 weeks after the second dose. Primary results from this study been published in Lyons et al. (2024).

#### 2.2.3 Psilodep

Psilodep was a comparative trial of the established SSRI drug escitalopram and psilocybin for the treatment of treatment-resistant major depressive disorder. The trial was a two-arm randomized controlled design, with 30 patients in the psilocybin group and 29 in the escitalopram group. Both groups participated in “daily dosing” as well as two “acute” treatment sessions separated by 3 weeks. The escitalopram group received a daily dose of 10 *mg* escitalopram that was doubled to 20 *mg* in the latter 3 weeks, while their acute doses of psilocybin were an inactive 1 *mg* placebo dose. The psilocybin group received a daily placebo capsule, while their acute doses of psilocybin were active 25 *mg* doses. Baseline outcome measures were acquired one day prior to the first acute session while follow-up responses were collected at 6 weeks. Acute experience measures were collected on the days of the acute sessions, with the maximum value from the two sessions being taken for analysis. Both groups received additional psychological support including an initial preparatory therapy session, supervision during and after the dosing, an integration session the day after the dosing, and a debriefing at the end of the study. The primary results of this study were published in Carhart-Harris et al. (2021), where it was found that psilocybin was at least as effective as escitalopram at reducing depressive symptoms.

### 2.3 Statistical analysis

All statistical analysis was carried out in R version 4.3.3 (R Core Team, 2024). The threshold for statistical significance was set at *P <* 0.05. For each sample, outcome measures were modeled with linear mixed effects models, which included fixed effects of time and drug condition, and random intercepts for participants. In the case of the Ceremony sample, only time was included as a fixed effect. Time was set with treatment contrasts, where baseline was the reference level, while drug condition was set with treatment contrasts in the case of a placebo condition (Insight) and effects contrasts in the case of an active comparison (Psilodep). Missing data was omitted pairwise during model fitting, such that participant data was only dropped for those groups from which it was missing. Quality of model fit was initially assessed through visual examination of residual and random effects distributions, and normality was tested using the Shapiro-Wilk test. All models conformed to assumptions. Model significance was assessed with F-tests for marginal analysis of deviance. *Post-hoc* contrasts were conducted via pairwise comparison of estimated marginal means, with Tukey's method for *P*-value adjustment. Effect sizes were calculated using Cohen's *d* for both the unadjusted empirical data as well as for the estimated marginal means; the latter is reported in tables. Associations between different outcome measures, as well as between acute experience measures and outcome measures, were assessed with Pearson correlations. Correlations were visualized with scatterplots fit with an OLS linear regression line. Comparison of correlations was performed using the Fisher z-test.

## 3 Results

### 3.1 Demographics

In the Ceremony study, 886 participants submitted data to the survey. Eight hundred and forty three completed the MLQ at baseline, although this number dropped to 399 at 4 weeks and 141 at the 6 month follow-up. Due to non-completion of the baseline assessment, demographic information was unavailable for 67 (7.6%) of the participants, so these are not included in the following statistics. 359 of the participants (40.5%) were female, 455 (51.4%) were male, and 5 (0.6%) reported other. The mean age was 44.4 +/– 12.6. Most participants were highly educated, with 693 (78.2%) having received a higher education. 743 (83.9%) of the participants were White, 12 (1.4%) were Black, 48 (5.4%) were Asian, 3 (0.3%) were American Indian or Alaska native, and 33 (3.7%) reported unknown or preferred not to say. While 330 (37.2%) participants had never used psychedelics previously, 489 (55.2%) had used them at least once in their life. Mental health status was mixed; while a majority of participants were clinically mentally healthy with 539 (60.8%) having no lifetime psychiatric diagnoses, 280 (30.6%) had at least one lifetime psychiatric diagnosis. Full demographic information can be found in Peill et al. (2022).

In the Insight study, 28 participants completed the MLQ following both 1 *mg* and 25 *mg* doses. Of these, 12 (42.9%) were female and 16 (57.1%) were male. Ages ranged from 28 to 59 years, with a mean of 40.6 +/– 8.7. The participants were mostly highly educated, with 16 (57.1%) having received a higher education. 24 (85.7%) were Caucasian, 1 (3.6%) was Black and 3 (10.7%) did not disclose their ethnicity. All participants had never previously used psychedelics. All participants were screened for good mental and physical health. Full demographic information can be found in Lyons et al. (2024).

In the Psilodep study, all 59 participants completed the MLQ after both dosing days. Of these, 20 (33.9%) were female and 39 (66.1%) were male. Ages ranged from 21 to 64 with a mean of 41.2 +/– 11.7. The majority of participants were highly educated with 45 (76.2%) having received a higher education. 52 (88.1%) were White. 43 (76.2%) had not used psilocybin previously, but no participants had previously used escitalopram. All patients had been diagosed with major depression prior to the study, and were also screened for moderate-to-severe depressive symptoms. For full demographic information, see Carhart-Harris et al. (2021). Tabulated demographic information for all samples can be retrieved in Supplementary Table S2.

### 3.2 Effects of psychedelics on the presence and search for meaning in life

To investigate the effects of psychedelics on the presence of and search for meaning in life, we compared scores on the MLQ-P and -S, assessed before and after a psychedelic experience, via linear mixed models across each of the three included studies: an online survey of attendees in naturalistic ceremonies (Ceremony), a laboratory study of psilocybin in healthy, psychedelic-naive participants (Insight), and a randomized clinical trial comparing psilocybin and escitalopram for depression (Psilodep).

#### 3.2.1 Presence of meaning

**Figure 1:**
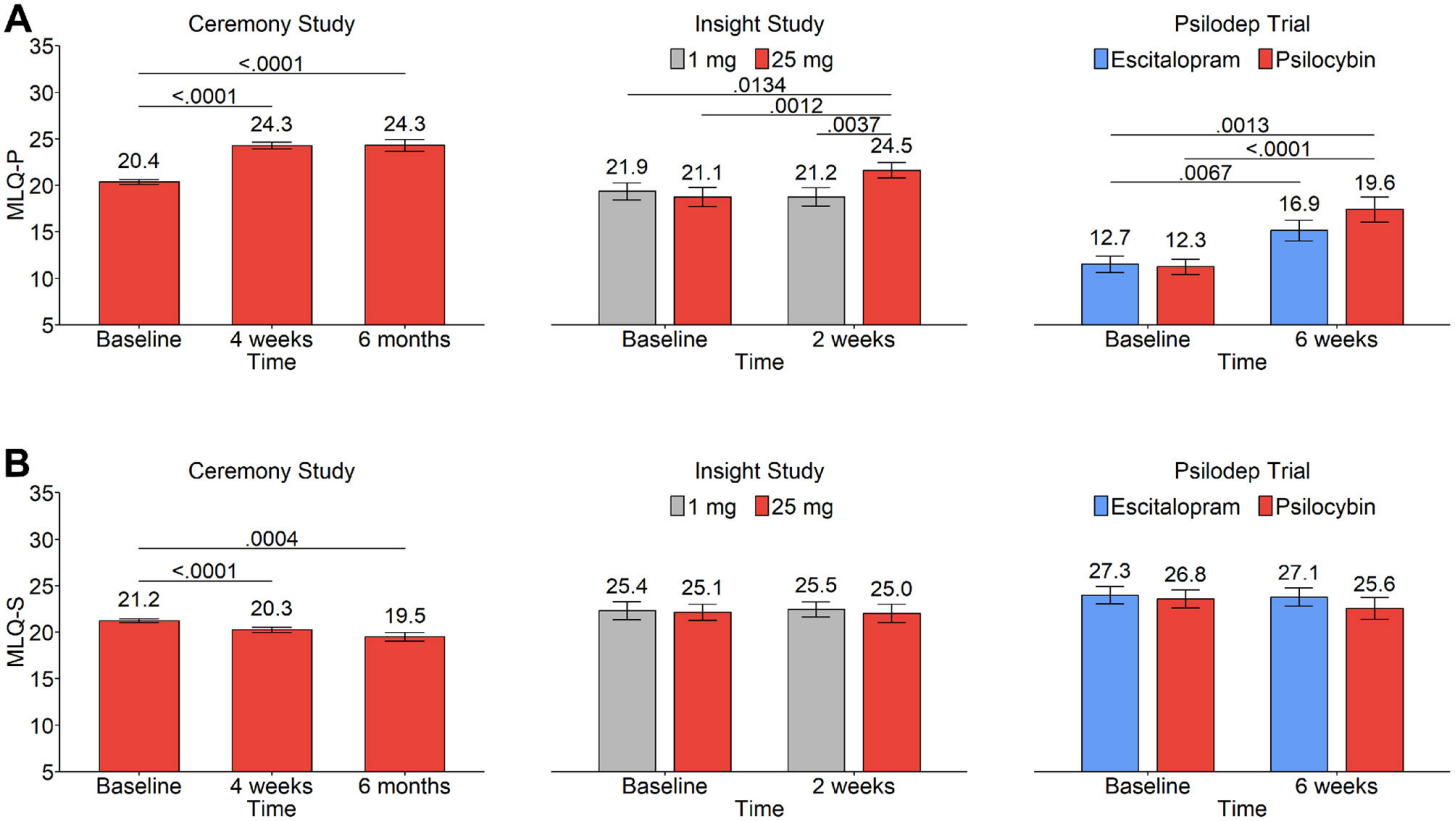
Scores on the MLQ before and after psychedelic use in three different studies: Ceremony study (left), Insight (center), and Psilodep (right). In all plots, bar height indicates the group mean and error bars indicate standard error. The group mean is also indicated by the value directly above each bar. Lines between bars indicate statistically significant group differences, as found from Tukey contrasts of mixed model marginal means, with the P-values indicated above the lines. **(A)** MLQ-P scores before and after psychedelic use in each study. **(B)** MLQ-S scores before and after psychedelic use in each study.

In the Ceremony sample, mixed effects modeling indicated a highly significant effect of time on the presence of meaning in life (*F*_2, 514_ = 79.85, *B*_4*weeks*_ = 3.54 [2.96 − 4.11 95% *CI*], *B*_6*months*_ = 3.14 [2.25 − 4.03 95% *CI*], *P <* 0.001, *n =* 843), suggesting substantial differences from before to after the psychedelic experience. Tukey contrasts of estimated marginal means confirmed that MLQ-P scores were significantly increased from baseline (20.4 [±7.8*SD*]) to 4 weeks after the ceremomy experience (24.3 [±7.3*SD*], *P <* 0.001, Cohen's *d* = 0.51), with no subsequent decrease even at the 6 months endpoint (24.3 [±7.4*SD*], *P* < 0.001, Cohen's *d* = 0.51 vs baseline, Figure 1A). In the Insight study, mixed modeling revealed a significant interaction of time*condition (*F*_1, 76_ = 9.72, *B*_2*weeks*:25*mg*_ = 4.03 [1.46 − 6.06 95% *CI*], *P =* 0.003, *n =* 28). Contrasts of estimated marginal means confirmed significant effects from baseline MLQ-P scores (21.1 [+/−6.3 *SD*]) to 2 weeks (24.5 [±4.9*SD*]) for the 25 *mg* condition, which were similar in magnitude to that observed in the Ceremony study (*P =* 0.0012, Cohen's *d* = 0.61, Figure 1A). Opposingly, no significant change over time was observed for the control 1 *mg* condition (*P =* 0.966), and there was also a significant difference between the 1 *mg* and 25 *mg* conditions at the 2 week endpoint (*P =* 0.0037, Figure 1A). Compared to the Ceremony and Insight studies, MLQ-P scores in the Psilodep study were 39.7% and 41.7% lower, respectively, at baseline in the psilocybin group (12.3 [±5.4*SD*]). Similarly low scores were also seen in the escitalopram group (12.7 [5.6±*SD*]). A mixed model indicated a trend level interaction effect of time*condition (*F*_1, 57_ = 2.92, *B*_6*weeks*:*psilocybin*_ = 2.99 [−0.52 − 6.50 95% *CI*], *P =* 0.093, *n =* 59), reflective of marginally greater increases in MLQ-P scores at 6 weeks following psilocybin (19.6 [±8.7*SD*]), compared to escitalopram (16.9 [±6.9*SD*]). Contrasts of estimated marginal means found significant increases in MLQ-P scores from baseline to 6 weeks in both the escitalopram (*P =* 0.007, Cohen's *d* = 0.69) and the psilocybin groups (*P <* 0.001, Cohen's *d* = 1.02, Figure 1A). All pairwise contrasts of estimated marginal means for MLQ-P can be found in Table 2. These results indicate that psychedelic use reliably enhances presence of meaning in life across contexts with varying levels of psychological support, but also that non-psychedelic treatments like escitalopram therapy can be associated with similar enhancements in the context of moderate-to-severe depression.

**Table 2:** Tukey contrasts of estimated marginal means (EMMs) from mixed effects models for MLQ-P scores.

**Sample**	**Contrast**	**EMM difference**	**Lower CL**	**Upper CL**	**df**	**t-ratio**	**Effect size**	***P-*value**
Ceremony	Baseline - 4 weeks	–3.54	–4.23	–2.85	514	–12.08	–0.46	**< 0.001**
Ceremony	Baseline - 6 months	–3.14	–4.21	–2.08	514	–6.94	–0.41	**< 0.001**
Ceremony	4 weeks - 6 months	0.39	–0.70	1.49	514	0.84	–0.05	0.676
Insight	Baseline 1 mg - Baseline 25 mg	0.71	–1.63	3.05	76	0.80	0.12	0.853
Insight	Baseline 1 mg - 2 weeks 1 mg	0.43	–1.98	2.83	76	0.47	0.07	0.966
Insight	Baseline 1 mg - 2 weeks 25 mg	–2.89	–5.32	–0.45	76	–3.12	–0.50	**0.013**
Insight	2 weeks 1 mg - Baseline 25 mg	0.29	–2.11	2.69	76	0.31	0.05	0.989
Insight	Baseline 25 mg - 2 weeks 25 mg	–3.60	–6.04	–1.17	76	–3.89	–0.63	**0.001**
Insight	2 weeks 1 mg - 2 weeks 25 mg	–3.32	–5.77	–0.86	76	–3.54	–0.58	0.004
Psilodep	Baseline Escitalopram - Baseline Psilocybin	0.32	–4.35	4.99	57	0.18	0.05	0.998
Psilodep	Baseline Escitalopram - 6 weeks Escitalopram	–4.24	–7.55	–0.93	57	–3.39	–0.63	**0.007**
Psilodep	Baseline Escitalopram - 6 weeks Psilocybin	–6.91	–11.58	–2.24	57	–3.92	–1.02	**0.001**
Psilodep	6 weeks Escitalopram - Baseline Psilocybin	4.56	–0.11	9.23	57	2.59	0.67	0.058
Psilodep	Baseline Psilocybin - 6 weeks Psilocybin	–7.23	–10.48	–3.98	57	–5.89	–1.07	**< 0.001**
Psilodep	6 weeks Escitalopram - 6 weeks Psilocybin	–2.67	–7.34	2.00	57	–1.51	–0.39	0.436

CL, confidence limit; df, degrees of freedom. Confidence limits are for 95%.

#### 3.2.2 Search for meaning

In the Ceremony sample, a mixed model revealed a significant effect of time on MLQ-S scores (*F*_2, 514_ = 12.54, *B*_4*weeks*_ = −0.94 [−1.39–0.49 95% *CI*], *B*_6*months*_ = −1.37 [−2.07–0.68 95% *CI*], *P* < 0.001, *n* = 843). This was reflected by a small yet significant decrease in MLQ-S scores from baseline (21.2 [± 5.3 *SD*]) to 4 weeks (20.3 [± 5.3 *SD*], *P* < 0.001, Cohen's *d* = −0.18), that was enhanced by 6 months (19.5 [± 5.63 *SD*], *P* < 0.001, Cohen's *d* = −0.33 vs. baseline, Figure 1B). See Table 3 for pairwise contrasts of MLQ-S estimated marginal means. In both the Insight and Psilodep samples, mixed models showed no significant interaction effects of time*condition on MLQ-S scores (Insight: *F*_1, 76_ = 0.16, *B*_2*weeks*:25*mg*_ = −0.44 [−2.67–1.78 95% *CI*], *P* = 0.692, *n* = 28; Psilodep: *F*_1, 57_ = 0.66, *B*_6*weeks*:*escitalopram*_ = 0.48 [−0.7–1.66 95% *CI*], *P* = 0.419, *n* = 59). There was also no significant main effect of time in either case (Insight: *F*_1, 76_ = 0.16, *B*_2*weeks*:25*mg*_ = −0.44 [−2.67–1.78 95% *CI*], *P* = 0.692, *n* = 28; Psilodep: *F*_1, 57_ = 0.66, *B*_6*weeks*:*psilocybin*_ = −0.48 [−0.70–1.66 95% *CI*], *P* = 0.419, *n* = 59). This result was reflective of very small decreases in MLQ-S scores over time in both studies. In Insight, the decrease in MLQ-S in the 25 *mg* condition from baseline (25.1 [± 5.5 *SD*]) to 2 weeks (25.0 [± 5.9 *SD*]) was very small (Cohen's *d* = −0.026, Figure 1B); similarly, in Psilodep, the decrease in the psilocybin group from baseline (26.8 [± 6.3 *SD*]) to 6 weeks (25.1 [± 7.5 *SD*]) was larger albeit still very small (Cohen's *d* = −0.18, Figure 1B). These results largely indicate that contrary to the effects on presence of meaning in life, psychedelic use may only produce very small decreases in the search for meaning in life, and the robustness of this effect varies with context. However, treatment with es*CI*talopram also did not decrease search for meaning in life in the context of moderate-to-severe depression, suggesting that this trait may be less amenable to pharmacological perturbation.

**Table 3:** Tukey contrasts of estimated marginal means (EMMs) from a mixed effects model for MLQ-S scores in the Ceremony study.

**Sample**	**Contrast**	**EMM difference**	**Lower CL**	**Upper CL**	**df**	**t-ratio**	**Effect size**	***P*-value**
Ceremony	Baseline - 4 weeks	0.94	0.40	1.48	514	4.11	0.18	**< 0.001**
Ceremony	Baseline - 6 months	1.37	0.54	2.21	514	3.87	0.255	**< 0.001**
Ceremony	4 weeks - 6 months	0.43	–0.43	1.29	514	1.18	0.08	0.467

CL, confidence limit; df, degrees of freedom. Confidence limits are for 95%.

### 3.3 Association of post-psychedelic changes in meaning in life with changes in wellbeing and depression severity

**Figure 2:**
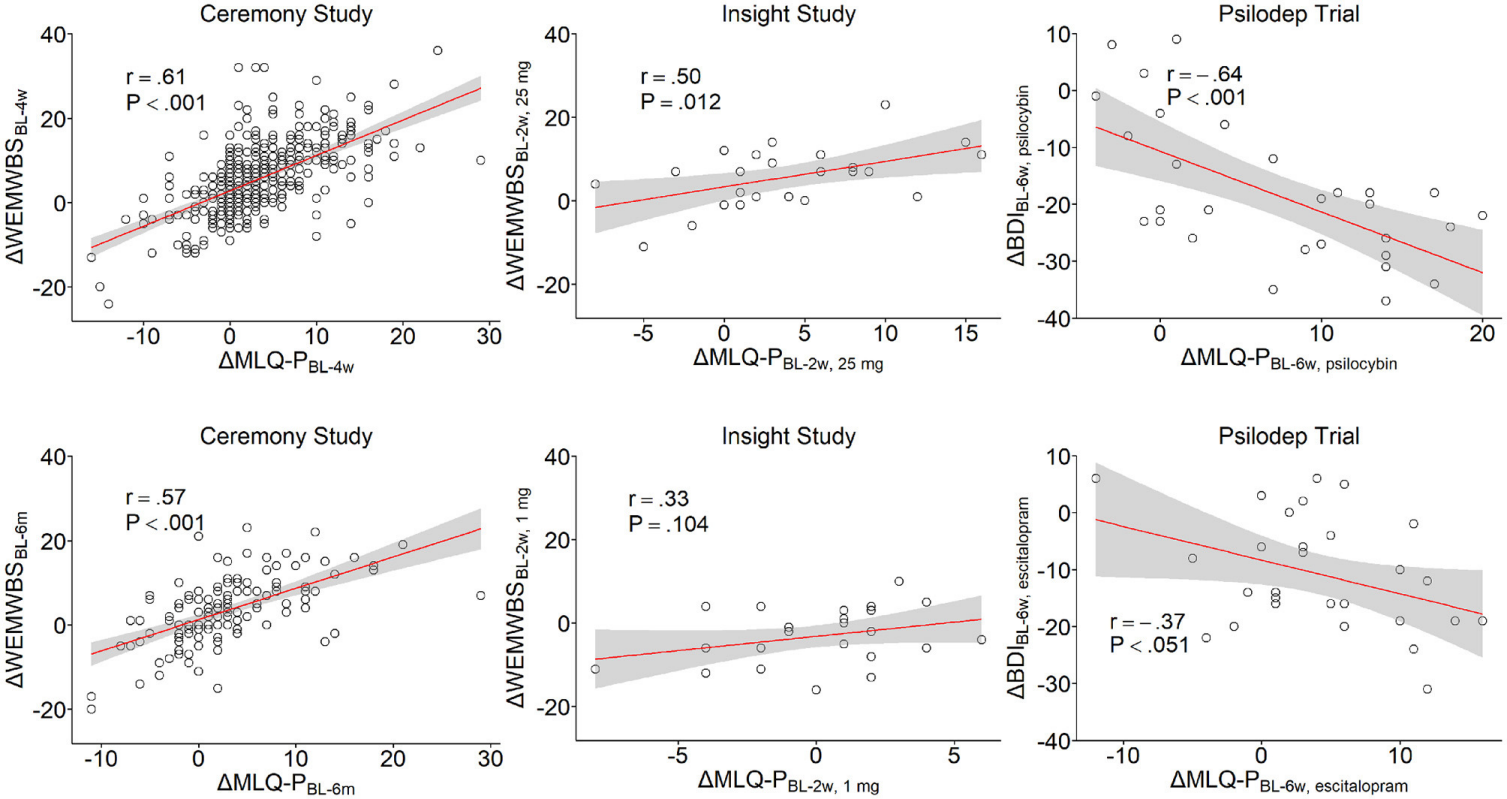
Correlations between changes in MLQ-P scores and changes in WEMWBS and BDI scores. Correlations with WEMWBS changes are reported for the Ceremony **(left)** and Insight samples **(center)**, while correlations with BDI changes are shown for the Psilodep sample **(right)**. In the Ceremony study, the correlations are shown for both the 4 week (top) and 6 month follow-ups **(bottom)**. In Insight study, correlations are shown for the 25 *mg* (top) and 1 *mg* conditions **(bottom)**. In the Psilodep trial, correlations are shown for the psilocybin **(top)** and escitalopram groups **(bottom)**. In each plot, points represent individual bivariate measurements, while the red lines show an OLS linear regression with the standard error indicated by the shaded region. Pearson's r and the corresponding P-value are also shown.

To investigate the relationship between psychedelic-induced changes in meaning in life and mental wellbeing, we examined the correlations between changes in MLQ scores and scores on the WEMWBS in the Ceremony and Insight studies. In previous results from both studies, psychedelic use was significantly associated with increased WEMWBS scores at the follow-ups compared to baseline (Kettner et al., 2021; Lyons et al., 2024). In the Ceremony sample, changes in MLQ-P scores were strongly positively correlated with changes in wellbeing at 4 weeks (*r* = 0.61, *P <* 0.001, *n =* 378,) and 6 months (*r* = 0.57, *P <* 0.001, *n =* 135, Figure 2). Conversely, there was a weak negative correlation between WEMWBS and MLQ-S score changes at 4 weeks (*r* = −0.27, *P <* 0.001, *n =* 378) and no correlation at 6 months (*r* = −0.06, *n =* 135, *P =* 0.485). A similar pattern was observed in the Insight study for the 25 *mg* condition, where changes in WEMWBS and MLQ-P scores at the 2 week endpoint were moderately positively correlated (*r* = 0.50, *P =* 0.012, *n =* 24, Figure 2), while a weak and non-significant negative correlation was observed for the MLQ-S subscale (*r* = −0.33, *P =* 0.112, *n =* 24). Conversely, in the 1 *mg* condition, no significant correlations were observed for MLQ-P changes (*r* = 0.33, *P =* 0.104, *n =* 25, Figure 2) or MLQ-S changes (*r* = 0.37, *P =* 0.067, *n =* 25) with changes in WEMWBS scores. These correlations suggest that while psychedelic-related increases in presence of meaning are clearly associated with improvements in wellbeing, decreases in the search for meaning are only slightly related to wellbeing enhancements, and this relationship may further weaken over time.

To examine whether changes in meaning in life were also related to depression severity, we calculated correlations for changes in MLQ scores and changes in BDI scores at the 6 week endpoint of the Psilodep study. In the original trial results, both escitalopram and psilocybin produced reductions in BDI scores, although this was slightly larger in the psilocybin group (Carhart-Harris et al., 2021). We observed a strong negative correlation between changes in MLQ-P and BDI scores in the psilocybin group (*r* = −0.64, *P <* 0.001, *n =* 28), which was only moderate and trending in significance for the escitalopram group (*r* = −0.37, *P =* 0.051, *n =* 28, Figure 2). However, fischer's z-test to compare the two correlation coefficients found no significant difference (*z* = −1.31, *P =* 0.191, *n =* 28). For the MLQ-S, we found only very weak and non-significant correlations with changes in BDI scores for both the psilocybin (*r* = 0.09, *P =* 0.633, *n =* 28) and the escitalopram group (*r* = 0.05, *P =* 0.802, *n =* 28). As with wellbeing, these correlations suggest that while increases in the presence of meaning in life following psychedelic use is associated with reduced depression severity, decreases in search for meaning do not relate to improvements in depression symptoms. Furthermore, we cannot soundly conclude that enhancements of meaning occurring after psilocybin-assisted therapy are more strongly related to depression improvements over and above meaning enhancement occuring from escitalopram treatment.

### 3.4 Acute psychedelic effects predicting post-psychedelic changes in meaning in life

**Figure 3:**
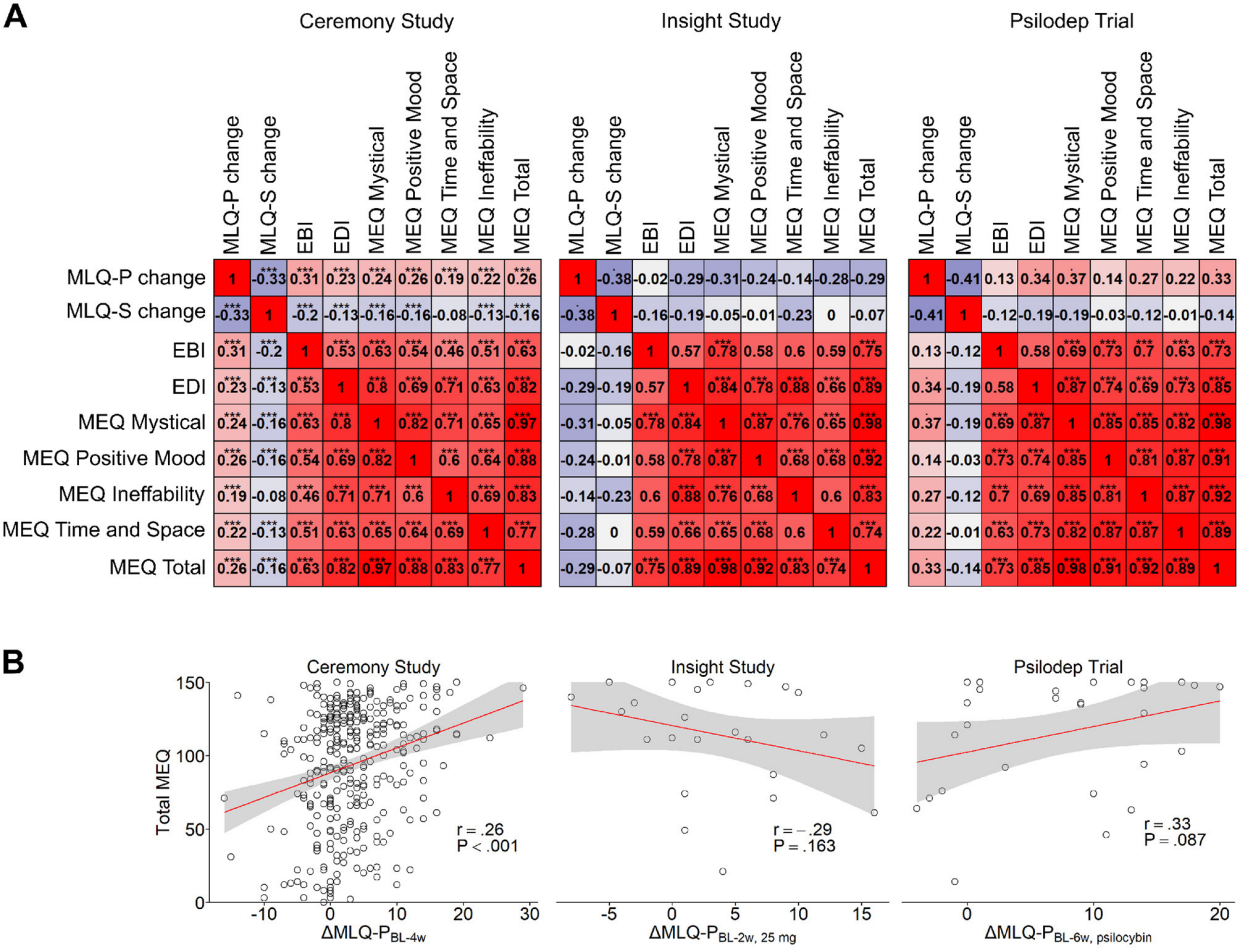
Correlations between acute experience measures and changes in MLQ scores. **(A)** Correlation matrices for changes in MLQ-P and MLQ-S scores and 7 acute experience measures: EBI, EDI, the 4 subscales of the MEQ and total MEQ scores. One matrix is shown for each study: Ceremony (left), Insight (center) and Psilodep (right). In each matrix, red values indicate positive correlations and blue values indicate negative correlations. The statistical significance is indicated by stars above each correlation coefficient, where “.” = *P <*0.1 and “***” = *P <*0.001. **(B)** Linear correlations between total MEQ scores and changes in MLQ-P scores before and 4 weeks after psychedelic use in the Ceremony study (left), 2 weeks after psychedelic use in the 25 *mg* condition of Insight study (center) and 6 weeks after psilocybin use in the Psilodep trial (right). Points represent individual bivariate measurements, while the red lines show an OLS linear regression with the standard error indicated by the shaded region. Pearson's r and the corresponding P-value are also shown in each case.

To determine if specific qualities of the psychedelic experience, including mystical-type experience, predicted subsequent changes in meaning in life, we examined correlations between acute experience measures and changes in MLQ scores. In the Ceremony sample, weak positive correlations were found between MLQ-P changes and EDI scores (*r* = 0.23, *P <* 0.001, *n =* 324), as well as more strongly with EBI scores (*r* = 0.31, *P <* 0.001, *n =* 324). Only weak positive correlations were observed between MLQ-P changes from baseline to 4 weeks and all subscales of the MEQ (Mystical: *r* = 0.24, *P <* 0.001; Positive mood: *r* = 0.26, *P <* 0.001; Time/space: *r* = 0.19, *P <* 0.001; Ineffability: *r* = 0.22, *P <* 0.001; *n =* 324 for all sub-scales, Figure 3B). Inverse relationships were observed for MLQ-S changes, which were negatively predicted most strongly by the EBI (*r* = −0.20, *P <* 0.001, *n =* 324), followed by the MEQ (Mystical: *r* = −0.16, *P =* 0.003; Positive mood: *r* = −0.16, *P =* 0.004; Time/space: *r* = −0.08, *P =* 0.153; Ineffability: *r* = −0.13, *P =* 0.023;*n =* 324 for all subscales) and EDI (*r* = −0.13, *P =* 0.017, Figure 3A). In the Insight sample, no statistically significant correlations between acute experience measures and changes in MLQ scores were found for the active 25 *mg* condition. All the correlations with MLQ-P scores were negatively signed, with the MEQ showing the strongest effect (Mystical: *r* = −0.31, *P =* 0.134; Positive mood: *r* = −0.24, *P =* 0.255; Time/space: *r* = −0.14, *P =* 0.507; Ineffability: *r* = −0.28, *P =* 0.171;*n =* 25 for all subscales, Figure 3B), followed by the EDI (*r* = −0.29, *P =* 0.161, *n =* 25) and the EBI (*r* = 0.02, *P =* 0.932, *n =* 25). For changes in MLQ-S scores, correlation coefficients were again negative, where the EDI showed the greatest effect (*r* = −0.29, *P =* 0.161, *n =* 25), followed by the EBI (*r* = −0.16, *P =* 0.439, *n =* 25, Figure 3A), and the MEQ (Mystical: *r* = −0.05, *P =* 0.811; Positive mood: *r* = −0.01, *P =* 0.975; Time/space: *r* = −0.23, *P =* 0.261; Ineffability: *r* = 0.00, *P =* 0.990; *n =* 25 for all subscales, Figure 3B). In the Psilodep sample, several weak-moderate positive correlations trending toward statistical significance were observed for changes in MLQ-P scores in the psilocybin group. The MEQ showed the strongest effect (Mystical: *r* = 0.37, *P =* 0.052; Positive mood: *r* = 0.14, *P =* 0.466; Time/space: *r* = 0.27, *P =* 0.170; Ineffability: *r* = 0.22, *P =* 0.265;*n =* 28 for all subscales, Figure 3B), followed closely by the EDI (*r* = 0.34, *P =* 0.075, *n =* 28), while the EBI showed only a small and non-significant effect (*r* = 0.13, *P =* 0.520, *n =* 28). A similar pattern of effect sizes was observed for correlations with the MLQ-S, however the signs were negative and showed no statistical significance. Of these, the MEQ showed the strongest effect (Mystical: *r* = −0.19, *P =* 0.338; Positive mood: *r* = −0.03, *P =* 0.898; Time/space: *r* = −0.12, *P =* 0.539; Ineffability: *r* = −0.01, *P =* 0.977; *n =* 28 for all subscales), followed by the EDI (*r* = −0.19, *P =* 0.338, *n =* 28) and then the EBI (*r* = −0.12, *P =* 0.557, *n =* 28, Figure 3A). These results provide some support for the hypothesis that mystical-type experiences positively predict greater enhancements of meaning in life. However, ego-dissolution and emotional breakthrough experiences may also provide similar benefits, and these effects may vary according to contextual factors.

## 4 Discussion

Meaning in life, although relatively under-researched (King and Hicks, 2021), presents a target for improving both mental and physical health that are independent from those conferred by other traits such as life satisfaction and wellbeing (Steger et al., 2006; McKnight and Kashdan, 2009; Hill and Turiano, 2014; Waytz et al., 2015). Due to their ability to produce rapidly transformative (Haijen et al., 2018; Mans et al., 2021) and spiritually-meaningful experiences (Griffiths et al., 2006, 2008; Gukasyan et al., 2022), psychedelic drugs like psilocybin have shown promise as potential enhancers of meaning (Hartogsohn, 2018; Griffiths et al., 2016; Nayak et al., 2023). The present study provided confirmatory evidence for this hypothesis, demonstrating that reported meaning in life increased from before to after psychedelic use across three distinct study samples, including naturalistic ceremonial psychedelic use (Ceremony), a laboratory study of healthy participants (Insight) and a clinical trial of psilocybin against escitalopram for patients with depression (Psilodep). Additionally, we found correlations between enhanced meaning in life and improvements in other outcomes, including wellbeing and depressive symptoms, as well as correlations with acute subjective psychedelic effects.

Significant increases in MLQ-P scores were observed following psychedelic use in all three samples, demonstrating that psychedelics can enhance meaning in life across a variety of environmental and psychological contexts. This is line with prior studies indicating increased MLQ-P ratings following psychedelic use (Mans et al., 2021; Timmermann et al., 2024), as well as with studies showing increased purpose in life (Griffiths et al., 2016) and reduced loss of meaning (Ross et al., 2021) in cancer patients, and increased meaning in the world (Nayak et al., 2023) after psychedelic use. The enhancement of meaning in life also parallels previous results involving changes in other constructs across similar contexts, including general wellbeing (Haijen et al., 2018; Kettner et al., 2021), connectedness (Watts et al., 2022), psychological insight (Peill et al., 2022) and depression severity (Carhart-Harris and Goodwin, 2017; Zeifman et al., 2020; Carhart-Harris et al., 2021). The magnitude of meaning in life enhancement was greatest in the Psilodep study, where presence of meaning at baseline was also lowest, thus potentially providing greater scope for improvements. This highlights psychedelics as potentially more effective for those with low levels of meaning in life and reiterates previous calls to tailour psychedelic therapy to those expressing certain psychological phenotypes (Zeifman et al., 2023). We also found that meaning in life was similarly enhanced for patients taking escitalopram, with no statistical significance between the groups. It is possible that psychosocial factors played a larger role in meaning enhancement than the type of drug, since both drug conditions saw a similar increase in meaning in life. However, a previous study specifically comparing these two groups found significantly greater MLQ score increases in the psilocybin group at 6 weeks, 3 months, and 6 months compared to the escitalopram group (Erritzoe et al., 2024). Therefore, given the numerically larger increase in the psilocybin group, it appears likely that the present analysis lacked the statistical power to reveal a group difference. It is worth noting that across studies, MLQ-P scores were lower than prior sample averages found in various populations including undergraduate students (Steger et al., 2006), US adults (Kobau et al., 2010), and even patients with life-threatening illness (Naghiyaee et al., 2020). This suggests that those who seek out a psychedelic experience—whether naturalistically, in research, or clinical trial settings—may have an attenuated sense of meaning at baseline. Therefore, the increases in presence of meaning observed here may be greater than those expected for the general population.

In regards to the search for meaning, we observed little impact of psychedelic intake on MLQ-S scores. Given the proposed relationship between heightened search for meaning and lower wellbeing (Steger et al., 2006, 2008; King and Hicks, 2021), we expected that MLQ-S scores would be decreased following psychedelic intake. However, statistically significant decreases were observed only in the Ceremony sample. While this may indicate that ceremonial settings can be a superior context for attenuating the desire to search for meaning, the observed effect sizes were still small and this result was more likely explained by the large sample size. Overall, it appears that psychedelics bear only a slight influence on the search for meaning. One explanation for this finding is that although psychedelics can increase the presence of meaning, this pharmacologically-assisted enhancement does not necessarily correlate to a reduced search for meaning as might occur with meaning-making through other life events. However, this result can also be reconciled with the generally positive effects of psychedelics (Haijen et al., 2018; Mans et al., 2021) by considering the complex nature of searching for meaning (King and Hicks, 2021). Provided it is not overly obsessive or inflexible, it appears reasonable that seeking out meaning could reflect an open and exploratory mindset with opportunities for learning and self-improvement. Indeed, while MLQ-S scores have been associated with anxiety and unhappiness, they have also been found to be related to curiosity, openness and absorption (Steger et al., 2008). Furthermore, some evidence suggests that day-to-day searches for meaning can predict its later acquisition within individuals (Newman et al., 2018). Considering the complex nature of the psychedelic experience, these findings could also be interpreted as indicating that psychedelic experiences can open as many existential questions as they answer. Although the extent of searching for meaning showed little change overall, it is possible that the context or methods of this search may be rendered more productive or fruitful by psychedelics. This hypothesis is supported by the observation of secondary decreases in MLQ-S scores at 6 months compared to 4 weeks in the Ceremony sample. Additional measures probing differences in individual approaches to the search for meaning in life and their relation to wellbeing would be needed to confirm this speculation (King and Hicks, 2021).

### 4.1 The impact of psychedelic-assisted meaning enhancement on mental health

Although the positive relationship between meaning in life and mental health has been detailed (King and Hicks, 2021), it was unknown if this would hold for meaning made following psychedelic use. Meaning made through pharmacological routes, which can acutely be disorganized and even delusional in nature (Carhart-Harris et al., 2016b), may be too illusory and fleeting to establish long-term associations with wellbeing (Hartogsohn, 2018). Therefore, we compared changes in MLQ scores to changes in other wellbeing measures, including general wellbeing via the WEMWBS and depression severity via the BDI. We found that in the Ceremony and Insight samples, changes in MLQ-P scores were robustly correlated with changes in WEMWBS scores, while in the Psilodep trial there was a strong negative relationship between meaning in life and depression severity. This shows that psychedelic-assisted changes in meaning are still strongly associated with other aspects of mental health across various contexts, and supports a mechanism for co-occurring enhancements of multiple facets of wellbeing following psychedelic use. It is worth noting that the correlations in each case were not very strong (0.5 < r < 0.64), indicating that meaning in life increases can occur in the absence of similarly sized increases in other wellbeing measures, which supports its status as a separate construct (Steger et al., 2006; King and Hicks, 2021).

A related observation from the longer timescale of the Ceremony study was that while MLQ-P scores remained at the same level from 4 weeks to 6 months post-ceremony, general wellbeing decayed over this same period. Accordingly, the strength of the correlation between MLQ-P and WEMWBS scores also slightly decayed over this period. On one hand, this could suggest that while meaning made through psychedelics is robust, its contribution to wellbeing is lessened over time. Alternatively, it might mean that psychedelic use enhances multiple facets of well-being, some of which decay over time, but some of which—including meaning in life—remain strengthened for longer. The latter hypothesis is supported by reports of the psychedelic experience as one of, or the, most meaningful of one's life, even after very long time periods (Griffiths et al., 2008; Johnson et al., 2017). This consistency reinforces the hypothesis that the psychedelic experience is embedded as a core memory in one's life narrative, from which meaning is consistently derived even if other aspects of mental health decline (Stoliker et al., 2022). In this way, psychedelic experiences could serve similar roles to meaningful religious experiences (Steger and Frazier, 2005), particularly in modern society where both religious commitment and sense of meaning are dwindling (Hartogsohn, 2018).

In the Psilodep sample, we saw some evidence for a stronger relationship between psychedelic-assisted meaning enhancement and mental health than for SSRI-assisted meaning enhancement. Although we did not observe a significant difference between the correlation strengths for the given sample size, the relationship for MLQ-P and BDI score changes in the escitalopram group was on the verge of statistical significance (*P =* 0.051) while for the psilocybin group it was highly significant (*P <* 0.001). It is important to note that all participants received substantial psychotherapy alongside both treatments, which likely contributed to the meaning enhancement observed in both groups (see below for a further discussion of context). Intriguingly however, this result points toward meaning enhancement from psychedelic therapy being more closely linked to mental health improvements than that from SSRI therapy. This could be related to the proposed differences in the mechanisms of psychedelic and SSRI therapies, namely that the former permits acutely intensive yet often beneficial emotional processing while the latter dampens emotions to improve resilience (Carhart-Harris and Goodwin, 2017; Hartogsohn, 2018). This mechanistic difference is likely related to differential outcomes for psychedelic use, such as a higher probablity of negative outcomes for those with emotional instability (Marrocu et al., 2024), as well as suggestions that psychedelic-assisted therapy works by reducing experiential avoidance (Zeifman et al., 2023). Under this mechanistic model, psychedelic therapy may produce more optimal wellbeing outcomes for those who lack a sense of meaning due to overly constrained patterns of cognition. However, due to the correlational nature of our analysis, we cannot conclude that meaning-making precedes wellbeing enhancement (or vice versa), nor can we rule out that the particular psychotherapeutic support received was key to any observed differences between treatment groups.

### 4.2 The role of context in psychedelic-assisted changes in meaning in life

A key strength of the present study was our integration of data from a selection of studies with a variety of environmental and psychological contexts, which was lacking in previous investigations of psychedelic-induced meaning change (Nayak et al., 2023), despite being key to the outcomes of psychedelic use (Carhart-Harris et al., 2018; Kettner et al., 2019; Marrocu et al., 2024). We can conclude that psychedelic use is associated with enhancements of meaning in life across these contexts, including naturalistic and controlled settings, and healthy or depressed psychological states.

Ample research has indicated that specific aspects of the acute psychedelic experience can shape subsequent outcomes. The most prominent of these acute states include the mystical-type experience (Haijen et al., 2018; Roseman et al., 2018; Weiss et al., 2024), the ego-dissolution experience (Barrett et al., 2016; Nour et al., 2016) and emotional breakthrough (Roseman et al., 2019; Watts et al., 2022; Weiss et al., 2024), which each predict more positive long-term outcomes and are also shaped by context (Carhart-Harris et al., 2018). To see if these were also related to psychedelic-assisted changes in meaning in life, we examined correlations between MEQ, EDI, and EBI acute experience scores with changes of MLQ-P and MLQ-S scores. Interestingly, while we observed some relationships between MEQ, EBI, and EDI scores and meaning in life, the results were not consistent across samples. In the Ceremony study, we observed the expected trends, whereby greater MEQ, EDI, and in particular EBI scores were associated with larger increases in MLQ-P scores and larger decreases in MLQ-S scores, which were similar in effect size to previous studies examining wellbeing (Haijen et al., 2018; Roseman et al., 2018). In the Psilodep study, the relationships were similar in magnitude but not quite statistically significant, possibly due to the reduced sample size. These results are in agreement with the prior studies and show that the quality of the acute psychedelic experience plays an important role in changes in meaning in life. However in the Insight study, no significant correlations were found, and the direction of the association was reversed for the MLQ-P. While the lack of significance could simply be due to the smaller sample size in this study, this disparity could also reflect contextual differences. For example, psychedelic therapy for depression is thought to benefit from a larger dose of 25 *mg* or more (Goodwin et al., 2022; Perez et al., 2023), which is concurrently more likely to induce mystical experiences (Ko et al., 2023). In contrast, healthy, psychedelic-naive participants may not benefit as strongly from a more intense experience, as they may be more likely to experience adverse effects such as anxiety due to their lack of experience. It is worth noting that across all the samples, the MEQ, EDI, and EBI scores were strongly positively correlated, suggesting that the scales were equally valid and properly interpreted in all contexts.

Individual differences in long-term responses to acute psychedelic effects may be related to their proposed mechanism of action. Recent neuropsychological models of psychedelic action suggest that psychedelics act to increase cognitive and neuronal flexibility (Daws et al., 2022; Singleton et al., 2022) to permit reframing of beliefs (Carhart-Harris and Friston, 2019; Zeifman et al., 2025), which can involve the discovery of new meaning (Fischman, 2019). It has been suggested that mystical experience is a subjective read-out of commonly observed acute psychedelic-related brain changes including DMN disintegration (Carhart-Harris et al., 2017; Gattuso et al., 2023), global integration (Tagliazucchi et al., 2016) and increased entropy (Carhart-Harris, 2018). Contrary to these acute effects, some evidence has shown greater within-DMN connectivity and DMN-limbic connectivity in the post-psychedelic phase (Carhart-Harris et al., 2017). Interestingly, the limited neuroimaging studies related to meaning in life bear some connection to these results. For example, increased resting state FC within the medial temporal lobe subsystem of the DMN has been found to predict a greater presence of meaning in life, but not scores on other related scales assessing life satisfaction (Waytz et al., 2015). A later study comparing meaning in life with loneliness further implicated the DMN, where higher scores on the Meaning and Purpose survey were associated with greater resting state FC within the DMN, stronger connectivity between default and limbic networks, as well as greater modularity within these two networks (Mwilambwe-Tshilobo et al., 2019). Following a model of psychedelic-induced belief change, a greater degree of mystical experience could therefore be more therapeutic for those with entrenched negative perspectives—particularly those regarding the self—that block the generation of meaning in life. Conversely, for healthy participants, a strong mystical experience may not be particularly helpful if they already have a positive sense of meaning in life and a good degree of cognitive flexibility. Nonetheless, the lack of an association between mystical experience and meaning in life observed here for the Insight sample does not undermine the overall enhancement in meaning in life still observed in this sample, nor the association of mystical experience with meaning enhancement observed in the other two samples.

In addition to the acute experience qualities, the environmental context in each study could play a role in enhancements of meaning in life. Importantly, we note that all the studies examined had some level of psychological support, which is a major factor for positive outcomes (Carhart-Harris et al., 2018), although this did vary between studies. In the Ceremony study, psychedelic experiences took place in groups and were led by a guide, allowing participants and the guide to support each other emotionally and foster a sense of community (Kettner et al., 2021). In the Psilodep trial, participants received substantial psychological therapy in addition to psilocybin treatment, and were also provided an emotionally supportive atmosphere during the experience itself (Carhart-Harris et al., 2021). Participants in the Insight study likely received the lowest level of support, although the experiences still took place in a therapeutic environment alongside supportive guides (Lyons, 2020; Spriggs et al., 2023). Therefore, from these results it is not possible to predict if similar enhancements of meaning in life would occur in other contexts lacking psychological support, such as naturalistic use in isolation. Furthermore, other aspects of the environmental context may have contributed to stronger increases in meaning in life. For example, the ritualistic and perhaps mystical setting of a psychedelic ceremony could have contributed to the further strengthening of the psychedelic experience as a powerful memory, while the clinical setting of the Psilodep trial could have pushed patients receiving psilocybin to further trust and believe the treatment would work. These contextual considerations are related to the suggestion that psychedelics may act as “placebo-enhancers,” whereby the profound subjective effects could produce an amplified placebo response, offering an explanation for their capacity to treat a wide variety of conditions (Hartogsohn, 2018).

Relatedly, participant characteristics and expectations in the different samples may have influenced subsequent changes in meaning in life. As previously mentioned, the Psilodep sample showed substantially lower MLQ-P scores at baseline compared to the other two studies. This may mean they had greater room for improvement, thus accounting for the large effect of psychedelic (and SSRI) therapy on MLQ scores. Additionally, their expectations about the outcome of the trial may have contributed to the improvement, particularly for the psilocybin group where patients could have been biased (Carhart-Harris et al., 2021; Szigeti and Heifets, 2024). However, a follow-up study found that while participants with higher suggestibility were more susceptible to psilocybin therapeutic response, levels of expectancy did not predict psilocybin response, despite it being greater for psilocybin than escitalopram (Szigeti et al., 2024). Likewise in the Ceremony study, participants seeking out a potentially life-changing experience could be more biased toward believing it would be positive; more specifically, participants seeking out a psychedelic ceremony of their own accord could have been more biased toward reporting favorable outcomes of psychedelic use. In both of these studies, a substantial proportion of the sample had previously used psychedelics, meaning they also had some prior expectations about how the drugs would affect them. However, the robust finding of enhanced meaning in life in the psychedelic-naive Insight sample improves confidence that psychedelics can improve meaning in life even when participants are unfamiliar with the subjective and long-term effects.

### 4.3 Limitations and future research

The nature of the samples used in this study presents several limitations. In addition to the potential confirmation bias and expectancy effects described in the section above, there may also be an effect of demand characteristics. Individuals signing up for a survey, study or clinical trial may be aware of the research surrounding psychedelics and the generally positive findings on wellbeing, and so are more likely to report positive effects. There was also a high level of attrition in the Ceremony study. In comparison to the 843 participants that completed the MLQ at baseline, only 141 (16.7%) completed it at the 6 month follow-up. It is possible that these remaining participants were more likely to report enhancements of meaning in life, while those who dropped out of the study may have had more difficult experiences. Although this cannot be ruled out in this study, previous investigations of attrition in observational studies of psychedelic use have found that baseline attitudes to psychedelics nor the intensity of acute challenging experiences predict participant dropout (Mans et al., 2021; Hübner et al., 2021); however, one study did find that younger age, low educational level and high conscientiousness were associated with increased attrition (Hübner et al., 2021), which is not ideal for study generalizability. Nonetheless, our finding of enhanced meaning in life across three separate samples increases the confidence in this result.

In addition to sample characteristics, our study is limited by use of strictly correlational analysis. While we observed some strong associations between meaning in life and other measures, we cannot infer the causal relationship between them. While it is tempting to say that enhanced meaning in life promotes wellbeing, it may well be that enhanced wellbeing facilitates the creation of meaning. Concurrently, while it is probably accurate to assume that mystical experiences promote meaning-making, we cannot rule out the possibility that individuals prone to make meaning are also prone to having mystical experiences when administered with psychedelics. Future studies should employ more structured experimental designs and mediation analysis to examine the directionality of the relationship between psychedelic-enhanced meaning in life and wellbeing, as has recently been done for psychological insight (Peill et al., 2022). A deeper understanding of the correlates and mechanism of psychedelic-enhanced meaning could also be bolstered with experience sampling and neuroimaging. Furthermore, our relative lack of analysis of both the psychological support and integration process (Bathje et al., 2022) of psychedelic-assisted therapy preclude a full understanding of how these drugs influence the meaning-making process. Future research should additionally focus on tightly controlling extra-pharmacological factors to delineate the contribution of the psychedelic experience itself to the perception of meaning (Hartogsohn, 2018).

### 4.4 Conclusions

Analysis of data from three studies has demonstrated that psychedelic use produces lasting enhancements in the perception of meaning in life. These enhancements were associated with positive changes in mental health, revealing meaning in life as another factor by which psychedelics can promote wellbeing. As with other psychological changes, contextual factors appeared to play an important role, with greater enhancement of meaning observed in psychedelic-assisted therapy for major depression. The extent of mystical experience, ego-dissolution, and emotional breakthrough were positively associated with meaning enhancement in two of the three studies, with the strength of the effects varying between contexts. Future studies should examine potential underlying mechanisms for the increased attribution of meaning, including the role of psychological support and the direction of causality between psychedelic-assisted meaning-making and general mental health.

## Data Availability

The data analyzed in this study is subject to the following licenses/restrictions: datasets can be made available for research purposes on request to the corresponding author of the primary studies. Requests to access these datasets should be directed to Hannes Kettner, hannes.kettner@ucsf.edu.
