# Supplementary material for: Enhanced meaning in life following psychedelic use: converging evidence from controlled and naturalistic studies
Source: Front Psychol. 2025 Jun 6;16:1580663. doi: 10.3389/fpsyg.2025.1580663 (PMC12179068; doi:10.3389/fpsyg.2025.1580663)
Supplement: Supplementary file 1 [file Data_Sheet_1.pdf]

Supplementary Material

1 SUPPLEMENTARY DATA

1.1 Tables

Item	Subscale
I understand my life's meaning.	Presence
I am looking for something that makes my life feel meaningful.	Search
I am always looking to find my life's purpose.	Search
My life has a clear sense of purpose.	Presence
I have a good sense of what makes my life meaningful.	Presence
I have discovered a satisfying life purpose.	Presence
I am always searching for something that makes my life feel significant.	Search
I am seeking a purpose or mission for my life.	Search
My life has no clear purpose.	Presence
I am searching for meaning in my life.	Search

Table S1. The Meaning in Life Questionnaire (MLQ) as used in the three studies. Items are rated from 1 (Absolutely untrue) to 7 (Absolutely true) giving a minimum score of 5 and a maximum score of 35 on each of the subscales. Item 9 is reverse-coded.

	**Ceremony**	**Insight**	**Psilodep**
Sample size	886	28	59
Gender (%)	Male: 51.4<br>Female: 40.5<br>Other: 0.6<br>NA: 7.6	Male: 57.1<br>Female: 42.9	Male: 66.1<br>Female: 33.9
Age (mean +/- SD)	44.4 ± 12.6	40.6 ± 8.7	41.2 ± 11.7
Education (%)	None: 0.7<br>High School or equivalent (GED): 7.0<br>Associate / Technical Degree: 6.5<br>College diploma: 28.2<br>Master's degree: 31.0<br>Doctorate or professional degree: 19.0<br>NA: 7.6	Secondary School Level: 42.9<br>University Level: 57.1	University level education: 76.3
Ethnicity (%)	White: 83.9<br>Black: 1.4<br>Asian: 5.4<br>American Indian / Alaska native: 0.3<br>Unknown: 1.2<br>Prefer not to say: 2.6<br>NA: 7.6	Caucasian: 85.7<br>Undisclosed: 10.7<br>Black: 3.6	White: 88.1
Previous psychedelic use (%)	No: 37.2<br>Yes: 55.2<br>NA: 7.6	No: 100	No psilocybin use: 72.9
Psychiatric history (%)	No diagnoses: 60.8<br>At least one lifetime diagnosis: 30.6<br>NA: 7.6	Healthy: 100	Moderate-to-severe depression: 100

Table S2. Demographic information for the three samples analysed in the study.
